# Design of a High-Gain Multi-Input LNA with 16.4 Degree Phase Shift Within the 32 dB Gain Range

**DOI:** 10.3390/s25061708

**Published:** 2025-03-10

**Authors:** Dong-Min Kim, Kyung-Duk Choi, Sung-Hwan Paik, Kyung-Jin Lee, Jun-Eun Park, Sang-Sun Yoo, Keum-Cheol Hwang, Youn-goo Yang, Kang-Yoon Lee

**Affiliations:** 1 Department of Electrical and Computer Engineering, Sungkyunkwan University, Suwon 16419, Republic of Korea; kdm2357@skku.edu (D.-M.K.); glyiiop@skku.edu (K.-D.C.); juneun.park@skku.edu (J.-E.P.); khwang@skku.edu (K.-C.H.); yang09@skku.edu (Y.-g.Y.); 2 SKAIChips, Suwon 16571, Republic of Korea; shback99@skaichips.co.kr (S.-H.P.); kjsj1221@skaichips.co.kr (K.-J.L.); syoo@skaichips.co.kr (S.-S.Y.)

**Keywords:** low-noise amplifier, multi-input, high gain, noise, multi-gain control, phase shift

## Abstract

This paper presents a high-gain multi-input low-noise amplifier (LNA) design aimed at achieving stable phase and minimal noise within a flexible gain range for modern wireless communication systems. The proposed LNA, designed using a CASCODE architecture and implemented in a 65 nm silicon-on-insulator (SOI) process, demonstrates significant improvements in isolation, noise reduction, and miniaturization. The SOI process reduces parasitic capacitance, enhancing performance and thermal/electrical isolation, critical for high-frequency applications. The CASCODE structure minimizes unwanted coupling between stages, enhancing signal integrity and maintaining stable operation across multiple gain modes. The LNA operates in the 2.3 GHz to 2.69 GHz frequency band and supports seven gain modes. It achieves a maximum gain of 21.45 dB with a noise figure of 1.03 dB at the highest gain mode. Notably, it maintains phase stability within 16.4 degrees across the entire gain range, ensuring consistent phase alignment, which is crucial for applications requiring precise signal alignment. The design eliminates the need for switching mechanisms typically used in conventional LNAs, which often introduce additional noise. This work demonstrates that the CASCODE-based multi-input LNA, implemented in a 65 nm SOI process, successfully meets the rigorous demands of high-frequency communication systems, achieving an optimal balance between gain flexibility, noise reduction, and stable phase control within a 32 dB gain range.

## 1. Introduction

With the rapid advancement of wireless communication technologies, the performance demands on low-noise amplifiers (LNAs) have significantly increased, particularly in terms of gain control, isolation, noise reduction, and phase stability. LNAs play a crucial role in RF front-end systems by amplifying weak received signals while maintaining a low-noise figure, thereby minimizing signal distortion and enhancing clarity [1]. High-performance LNAs are indispensable for high-speed data transmission applications, including cellular networks, satellite communications, and IoT devices, where maintaining superior signal quality is paramount [2]. Mobile devices are designed to support the previous generation’s technology, and when a new generation is introduced, the frequency band of the new device encompasses both the previous and current generations [3]. Both performance and structural improvements are required compared to previous generations, which ensures compatibility with emerging frequency bands. Therefore, receivers such as low-noise amplifiers (LNAs) must be capable of supporting multiple frequency bands [4]. Additionally, when the mobile device is located far from the signal source and the received signal strength at the RX antenna is extremely weak, the low-noise amplifier (LNA) must amplify the signal with maximum gain while maintaining a minimal noise figure (NF) [5]. Consequently, the design of LNAs capable of delivering high gain, low noise, and stable phases across multiple modes and input sources poses considerable challenges, particularly at higher frequencies.

This paper presents a high-gain multi-input LNA design that focuses on achieving a stable phase within a 16.4-degree range across seven gain modes, all within a 32-dB gain range. Utilizing a CASCODE structure and implementing a 65 nm Silicon-On-Insulator (SOI) process, the design takes advantage of improved isolation and noise performance. The SOI process improves amplifier performance by reducing parasitic capacitance. It also provides superior thermal and electrical isolation. The CASCODE structure inherently provides better isolation by minimizing unwanted coupling between the input and output stages, maintaining signal integrity, and preventing intermode interference. This makes CASCODE-based multi-input structures an optimal choice for managing multiple gain modes while minimizing noise interference. To further optimize performance, the layout design carefully considers parasitic capacitance and metal line inductance. Considering these parasitic factors in the layout process ensures that the LNA maintains high performance under various operating conditions, as these factors can have a significant impact on gain stability and phase control at high frequencies.

The proposed LNA operates within the frequency range of 2.3 GHz to 2.69 GHz and supports seven distinct gain modes. These modes allow for adaptive gain control under different signal conditions, achieving a maximum gain of 21.45 dB with a noise figure of 0.7 dB in the highest gain mode. An important feature of this design is its ability to maintain phase stability within 16.4 degrees across all gain modes, providing reliable performance for applications that require consistent signal alignment. Phase stability is especially important in phase-sensitive applications, as fluctuations can negatively impact overall system performance. When the gain mode changes, if the phase alignment between the end of one waveform and the beginning of the next repetition is not maintained, signal distortion may occur.

Traditional LNA designs often suffer from high noise levels due to the integration of switches for gain control, which adds parasitic elements and degrades the signal. In contrast, the CASCODE-based multi-input approach used here eliminates the need for switches, effectively lowering noise and improving linearity. Through careful selection of this architecture and careful layout considerations for the parasitic components, the design achieves an optimal balance between gain flexibility, noise performance, and phase stability. This paper shows how the CASCODELNA design using the 65 nm SOI process through the proposed architecture ensures high-gain multiple inputs while meeting the complex requirements of modern high-frequency communication systems.

## 2. Proposed Architecture and Technique

**Figure 1:**
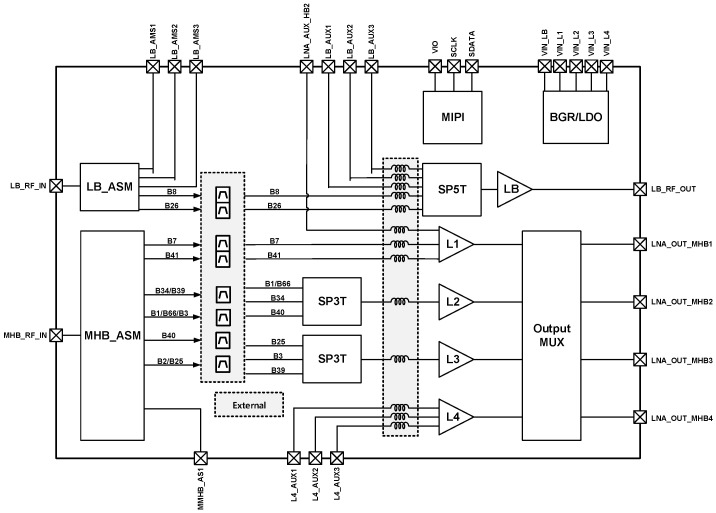
Top diagram.

Figure 1 is a top block diagram of the proposed system, which consists of 5 LNAs, MIPI, and a switch. By receiving the signal from the antenna and controlling it with MIPI, the user can use the desired LNA according to the desired frequency band and gain. The inductor is a high Q factor from Murata.

Figure 1 presents the overall top-level diagram along with a research paper on the design of the low-noise amplifier (LNA), referred to as L1. A conventional LNA structure typically employs a common-source configuration with a single input and a single output. In this design, a switch is placed at the input stage, allowing the user to select the desired frequency band from multiple available bands. However, since noise is a critical factor in LNA performance, the presence of a switch in the signal path inevitably introduces additional noise, creating unfavorable conditions for achieving high-performance LNA design.

To address this limitation, this paper proposes a multi-input structure with three inputs and one output. By assigning a dedicated LNA core to each frequency band, this approach eliminates the need for a switch in the signal path, thereby preventing the noise that would otherwise be generated. As a result, the proposed structure effectively improves the overall performance of the LNA

**Figure 2:**
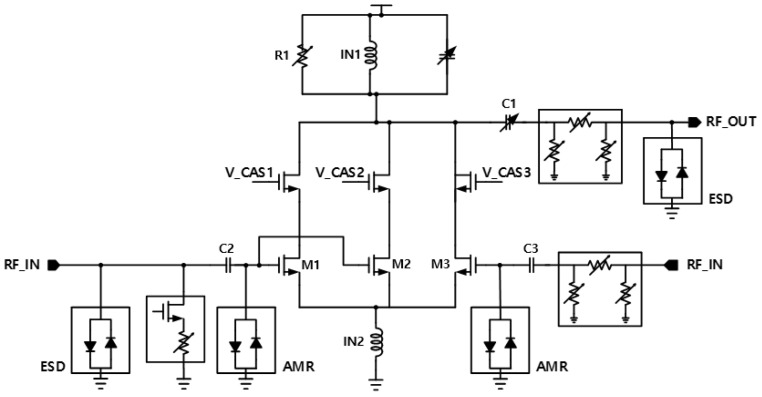
Structure of the designed LNA Schematic.

In the structure shown in Figure 2 below, in the general structure, the mode is changed according to the band using a switch to change the appropriate band, but in the multi-input structure, the core stage is placed separately for each band, and the band and mode are changed through a digital signal.

The mode is divided by gain step with a total of 7 steps, and the current consumption is from the maximum gain mode of 14.3 mA to the minimum gain mode of 2 mA.

To prevent phase variations caused by mode switching, input and output phase blocks were implemented in the design.

Table 1 illustrates the dimensions of the elements shown in Figure 2. The gate bias of the CASCODE stage is set to 1 V, and all MOSFETs are designed as body-contact devices.

The circuit includes ESD diodes and AMR diodes. The ESD diodes are designed to provide instantaneous overvoltage protection for the input and output, safeguarding the circuit from damage caused by electrostatic discharge. The AMR diodes are implemented to prevent the destruction of the input MOSFET device by mitigating the effects of high voltage applied to the input.

When designing a low-noise amplifier (LNA), the most critical factors to consider are the noise figure (NF) and gain. These parameters must be optimized to ensure that the input signal is transmitted with maximum accuracy within the desired frequency band. To achieve a low minimum noise figure (NFmin), the device impedance must be matched to the optimal noise impedance. Similarly, to achieve a high maximum stable gain (MSG), the input impedance must be matched to ensure efficient signal transmission. However, there exists a trade-off between these two parameters. Therefore, the design process must be carried out in accordance with the specified design requirements to achieve optimal performance.

First, by incorporating an external inductor at the input stage, the matching frequency can be adjusted with ease while preserving the S11 trajectory on the Smith chart. A common-source (CS) stage with an inductive generation structure was used in consideration of S11 matching, NF performance, and high-power performance [5]. Although source degeneration can lead to gain degradation, it is crucial to minimize the matching elements to ensure adequate gain, as this simplifies achieving S11 matching [6]. Additionally, the parasitic capacitance of Cgs in the input MOSFET adversely affects the gain, making it essential to minimize the layout size to mitigate this impact.

Also, a series capacitor is connected to the input stage to select the gain mode. Normally, the attenuator only controls the gain, but if it does, the phase will be distorted, so the series capacitor inside the input phase control block plays a big role in phase stability through optimized matching by mode.

Pi-type attenuators exhibit resistive characteristics (R), and as the input signal passes through the resistance, its phase changes due to interactions with the inductive or capacitive elements at the input of the low-noise amplifier (LNA). When the input impedance of the LNA is expressed as a complex number(jX), the phase variation can be described as shown in Equation (1):

(1) $$\theta =\tan^{-1}\left(\frac{X_{in}}{R_{in}+R_{s}}\right)$$

Rs = added series resistance.

Rin = real part of the LNA input impedance.

Xin = reactive part of the LNA input impedance.

**Figure 3:**
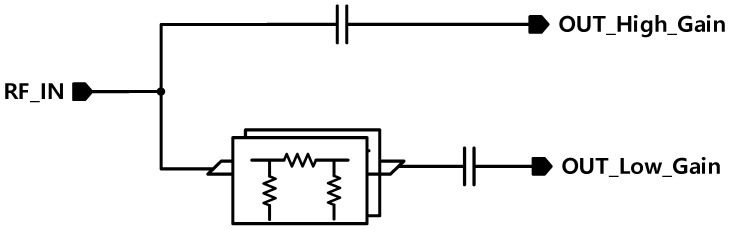
Input phase control block.

Therefore, a series capacitor is employed to compensate for the varying phase, minimizing the phase change effectively. See Figure 3.

In order to prevent the phase from shifting while changing the mode, a series capacitor is attached to prevent the phase from shifting by matching the phase that changes while passing through the LNA Core stage in high-gain mode and low-gain mode through the capacitor control by mode.

At the output stage, S22 is mainly controlled, and the most influential load inductor is located in parallel. The larger the load inductor, the higher the gain can be secured, but the center frequency is shifted to a lower frequency. Therefore, it is recommended to make the load inductor as large as possible to secure high gain within the scope of securing the area and S22 in the specified band.

The parallel capacitor also plays a role in shifting the S22 graph. Thirdly, the load resistance is connected in parallel, and a MOSFET is used as a switch to control the resistance through digital control by configuring a combination of resistors, thereby adjusting both gain and linearity. Additionally, at the output stage, an attenuator and a series capacitor are integrated to form a phase control block. Similar to the input stage, this block also functions as a DC block, playing a crucial role in preventing phase shifting and guaranteeing stable signal transmission.

**Figure 4:**
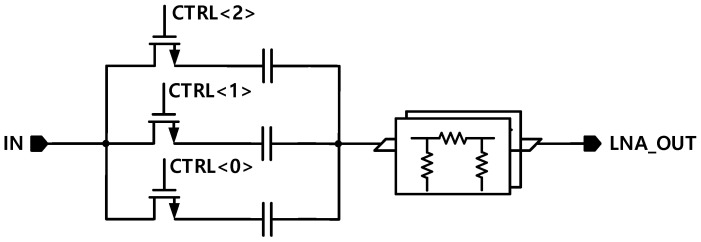
Output phase control block.

The output phase control block in Figure 4 enables adjustment of the distorted phase for each gain mode after the signal passes through the input, LNA core, and load stages. This adjustment is achieved using mode-specific series capacitors connected to the attenuator. Based on this approach, an LNA was designed with a wide gain range of 32 dB, encompassing 7 gain modes, ensuring that more than 20 dB of gain remains distortion-free.

## 3. Layout

**Figure 5:**
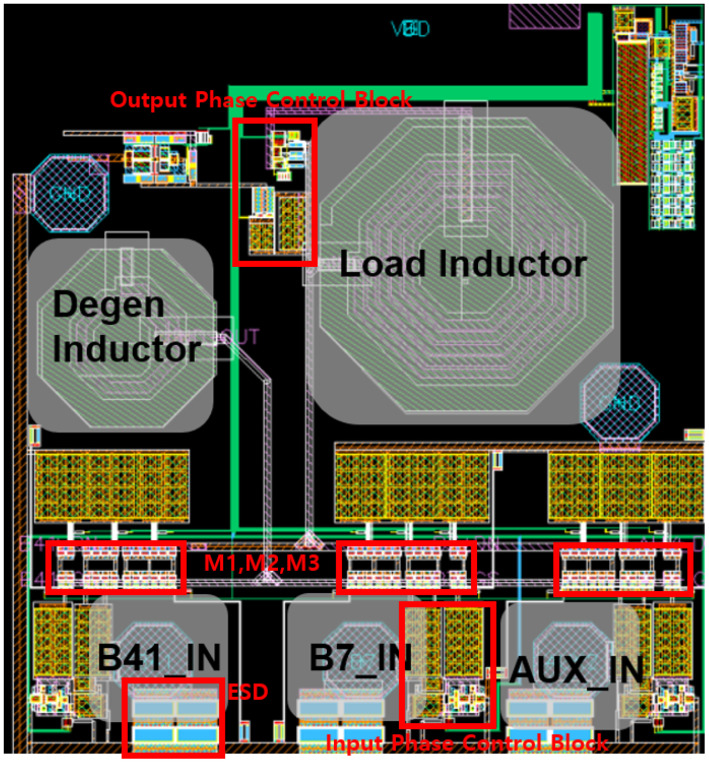
Designed LNA Layout.

When designing an LNA, the layout holds greater significance than the schematic. RF blocks, which are highly sensitive to parasitic component effects, necessitate meticulous attention to achieve the desired performance. During the LNA layout process, the visibility of Cgs parasitics on the input MOSFETs becomes increasingly critical, emphasizing the need to minimize their impact on gain. Additionally, in laying out the LNA core MOSFETs, stacking metal layers was deliberately avoided to reduce the prominence of parasitic components, see Figure 5.

## 4. Simulation and Measurement Results

**Figure 6:**
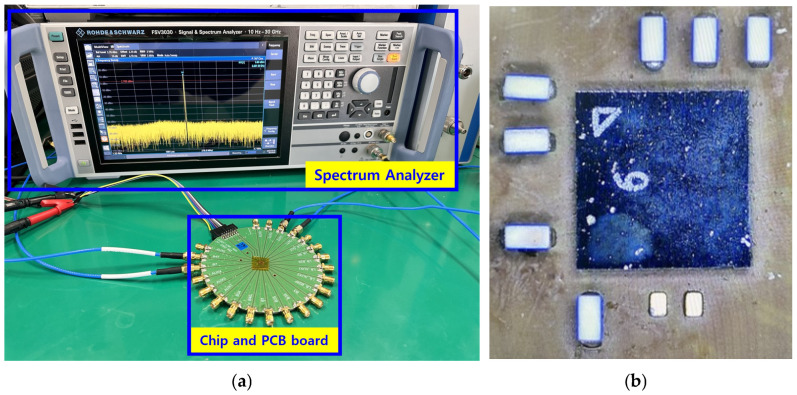
(**a**) Chip attached to the PCB board and measurement environment with the spectrum analyzer. (**b**) Chip photo and surrounding external inductor.

Figure 6a shows the measurement environment through the spectrum analyzer by attaching the chip to the PCB board. After connecting the LNA input and RF signal generator to the spectrum analyzer, the signal and power in the desired frequency band are about −30 dBm to −50 dBm, and the signal output is measured through the spectrum analyzer to check the output power and measure the LNA gain using the two power differences. Noise can also be calculated by putting a signal through the noise source and checking the output power. In order to reduce the loss by cable and PCB when proceeding, the loss of the signal was reduced through de-embedding.

Figure 6b shows the photo of the chip and the external inductor around it.

**Figure 7:**
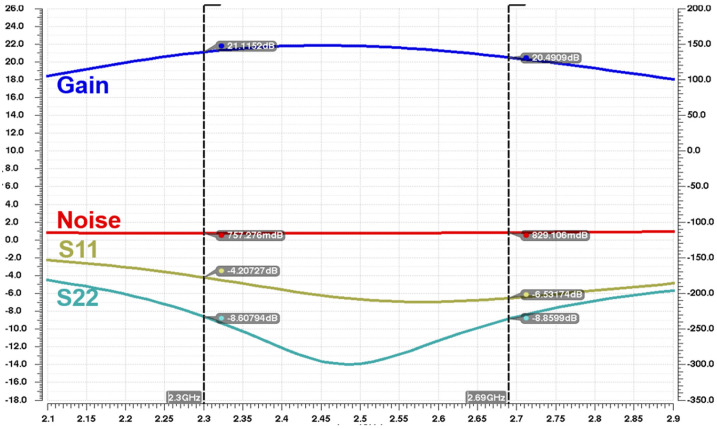
Gain, noise, S11, and S22 waveforms as post-simulation.

Figure 7 shows the post simulation results including the optimized layout and the laminate component of the board, and the maximum gain of 22.5 dB and noise of 0.7 dB were confirmed. In the 2.3 GHz~2.6 GHz band, the center frequency of S11 is −7 dB and the value of S22 is about −13 dB.

**Figure 8:**
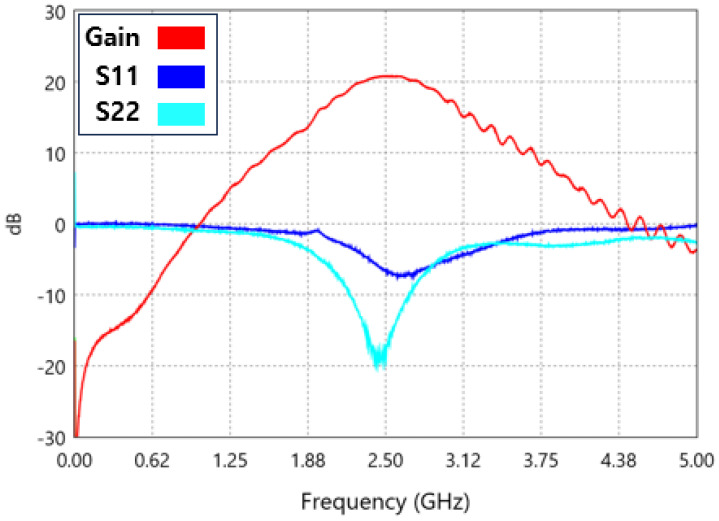
Gain, S11, and S22 waveforms as measured.

A comparison of the measured results with the simulation results in Figure 8 shows that the gain is 21.45 dB, S11 is −7.498 dB, and S22 is −19.784 dB. While the gain is approximately 1 dB lower than the simulation, the performance of the S11 and S22 graphs is improved. Notably, the S22 graph is shifted to the left due to the influence of parasitic components.

The peak S22 frequency remains nearly identical, while the peak S11 graph is observed to shift to the right. Similarly, the peak gain is also shifted to the right, appearing at 2.52 GHz.

**Figure 9:**
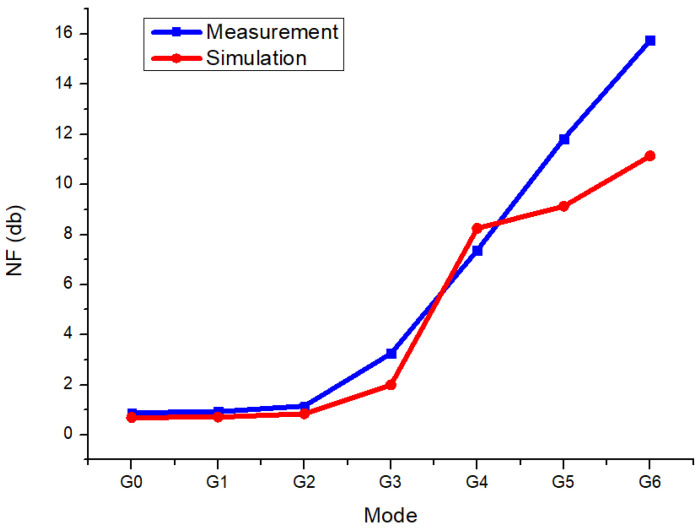
Measurement and simulation noise figure comparison.

Figure 9 presents a comparison of the noise figure (NF) results obtained from simulation and measurement. Except for the G4 mode, the measured NF values were higher than the simulated ones. This discrepancy is attributed to the parasitic components of both the chip and the measurement board, which were reflected in the measured results.

**Figure 10:**
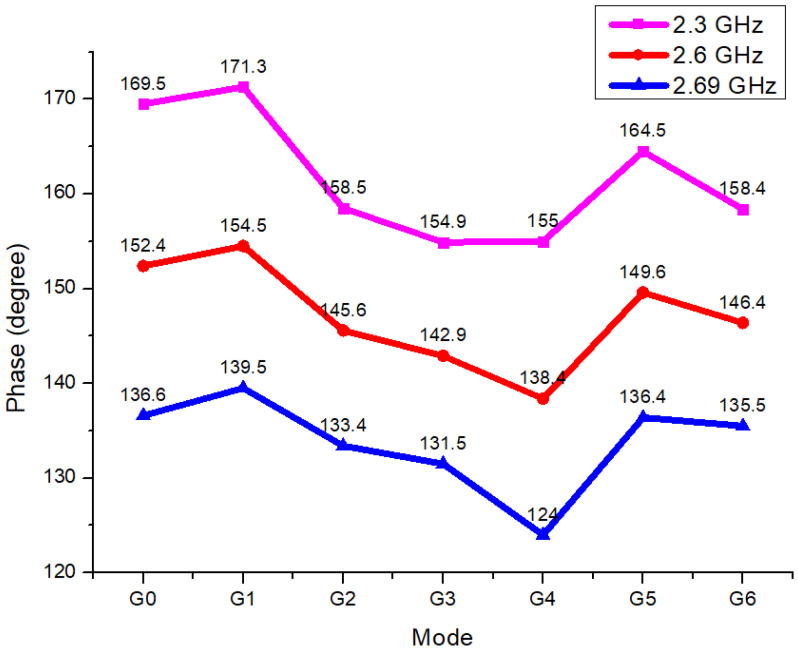
2.3 GHz~2.69 GHz phase measurement result.

When switching modes by frequency band using Figure 10, the PHASE measurement results vary, reaching a maximum deviation of 16.4 degrees within the 2.3 GHz to 2.69 GHz range.

Table 2 shows the LNA measurement performance by gain mode. One can see that the gain step changes by mode and the current and IIP3 performance changes.

Table 3 summarizes the performance and compares it with other works [4,5]. The LNA of this paper realizes the multi-gain mode with the lowest supply voltage of 1 V in the range of 2.3 GHz–2.69 Ghz, while achieving a high gain of 21.45 dB and good performance matching of S11 = −7.498, S22 = −19.784, and is designed to protect the chip through the implementation of the same HBM ESD protection circuit as the mass-produced chip.

When comparing [7,8], which have the most similar frequency band to this paper, it is observed that this work achieves the highest gain and the lowest NF. Additionally, when compared to [9], it exhibits a similar gain but has a higher NF and supply voltage than this research.

## 5. Conclusions

This paper presents the design of a high-gain multi-input LNA optimized for stable phase performance and minimal noise across a flexible gain range, tailored to modern wireless communication systems. The proposed LNA, developed using a CASCODE structure and fabricated with a 65 nm silicon-on-insulator (SOI) process, demonstrates notable improvements in isolation, noise suppression, and miniaturization. The SOI process effectively reduces parasitic capacitance, enhancing performance and electrical isolation, which are essential for high-frequency applications. The CASCODE configuration minimizes unwanted inter-stage coupling, enhancing signal integrity and ensuring stable operation across multiple gain modes.

Operating in the 2.3 GHz to 2.69 GHz frequency band, the LNA supports seven gain modes, reaching a maximum gain of 21.45 dB with a noise figure of 1.03 dB in the highest mode. Importantly, it maintains phase stability within 16.4 degrees across all ranges and gain modes, guaranteeing that phase consistency is maintained as gain is varied. This phase stability is essential for applications requiring consistent signal alignment and is an advantage over traditional LNAs that introduce noise through switching mechanisms for gain control.

By carefully addressing parasitic capacitance and metal line inductance in the layout, the design maintains high performance and reliability across operating conditions. This paper therefore demonstrates how the CASCODE multi-input LNA using the 65 nm SOI process effectively meets the stringent requirements of high-frequency systems, achieving an optimal balance between gain flexibility, noise minimization, and stable phase control within a 32 dB gain range.

## Figures and Tables

**Table 1:** Block device size.

	Size	Unit		Size	Unit
R1	25~550	Ω	C2	800	fF
M1	120/60	um/nm	C3	1.6	pF
M2	80/60	um/nm	IN1	2.88	nH
M3	40/60	um/nm	IN2	207	pH
C1	300~760	fF			

**Table 2:** Measurement performance by gain mode.

Mode	Gain (dB)	NF (dB)	IIP3 (dBm)	Current (mA)
G0	21.45	1.03	−6.08	14.53
G1	16.44	1.05	−4.10	14.54
G2	12.77	1.33	−0.47	12.19
G3	7.78	3.29	1.28	9.91
G4	0.52	7.33	2.70	2.79
G5	−4.30	11.80	4.79	1.95
G6	−9.13	15.78	6.21	1.97

**Table 3:** Performance summary and comparison.

Parameter	This Work	[7]	[8]	[9]	[10]	[11]	[12]	[13]
Freq. (GHz)	2.3–2.69	2.4–2.5	0.7–2.7	4–11.5	0.3–3.5	0.1–3.4	0.6–3.15	0.02–4.5
NF (dB)	1.03	1.3	2.95–3.4	2.75	2.9–3.5	3.4	<3.1	3.2–5.4
Gain (dB)	21.45	14.5	15.6–18.6	21	14.6	18.2	20.2	11.2–20.4
S11 (dB)	−7.498	−9	<−10	<−10	-	<−10	-	-
S22 (dB)	−19.784	−23		-	-	-		-
Multi-Gain	Yes	No	No	No	No	No	No	No
Supply Voltage	1	3.3	1.8	1.2	1.6	1.2	1.5	1.2
ESD	HBM	HBM	-	-	-	-	-	-
Tech. (nm)	65 SOI.	-	180	65	180	130	180	65

## Data Availability

The original contributions presented in the study are included in the article, further inquiries can be directed to the corresponding author.
